# An Extracellular Vesicle (EV) Paper Strip for Rapid and Convenient Estimation of EV Concentration

**DOI:** 10.3390/bios15050294

**Published:** 2025-05-06

**Authors:** Gisela Ströhle, Rebecca Goodrum, Huiyan Li

**Affiliations:** School of Engineering, University of Guelph, Guelph, ON N1G 2W1, Canada; gstrohle@uoguelph.ca (G.S.); rgoodrum@uoguelph.ca (R.G.)

**Keywords:** extracellular vesicles (EVs), EV concentration, paper-based immunoassay, fluorescence detection, biosensor strip

## Abstract

Extracellular vesicles (EVs) have emerged as promising biomarkers and therapeutic agents, yet their quantification remains technically challenging due to the limitations of conventional methods. Here, a low-cost, fluorescence-based, paper-strip immunoassay is presented for rapid and semi-quantitative estimation of EV concentration, inspired by pH strips. The assay utilizes nitrocellulose membranes functionalized with capture antibodies (anti-CD63, CD9, CD81) and fluorescent dye (ExoBrite™) for EV detection. Systematic optimization of assay parameters—including dye application sequence, incubation time, antibody configuration, and dye concentration—revealed that labeling EVs with dye and incubating on the nitrocellulose paper strips for 20 min yielded the strongest and most reproducible signal. A 200× dilution of ExoBrite™ dye was determined to provide the best balance between sensitivity and specificity. A standard curve generated through twofold serial dilution of EVs from ovarian cancer cell culture medium confirmed a positive, concentration-dependent fluorescence response, establishing a usable dynamic range. Compared to existing technologies, this platform enables fast, simple-to-implement EV quantification using minimal sample volume and equipment. The simplicity and scalability of the method offer strong potential for use in clinical diagnostics and EV research applications.

## 1. Introduction

Over the past decade, extracellular vesicles (EVs) have emerged as highly promising tools for diagnosing, therapeutics, and drug delivery systems [1,2,3]. These lipid bilayer-surrounded nanovesicles, which are secreted from nearly all living cells, exhibit heterogeneity in structure, content, function, and origin [4,5,6]. The release of EVs is a crucial process for removing unwanted compounds and maintaining cellular homeostasis [7,8]. Beyond their role as waste transporters, EVs serve as natural delivery systems, facilitating the transfer of metabolites, proteins, nucleic acids, and lipids between cells, thereby enabling communication both in the vicinity and over long distances within the extracellular environment [9,10,11,12]. EVs have been implicated in processes such as cancer progression, immune responses, viral pathogenesis, and diseases of the central nervous system [1,13,14,15].

EVs have gained attention as potential biomarkers for various diseases, as they have been shown to reflect the condition of their originating cells [16,17]. Research indicates a connection between EV concentration and content with conditions such as cardiovascular disease [17], liver disorders [18], and cancer [1,19]. The quantity of EVs tends to increase during disease, making them valuable biomarkers for detecting and tracking cancer progression [16,19]. Since EVs are present in various biological fluids (blood, urine, etc.) and cell culture supernatant, they provide a non-invasive means for evaluating disease progression, the response to a treatment, or providing initial indications of a pathology [11,16]. Besides therapeutic and diagnostic applications, accurate quantification of EVs guarantees reliability and reproducibility in experiments [20]. Identifying the number of EVs secreted under different conditions allows researchers to normalize the content of nucleic acids, proteins, and lipids per vesicle, offering insights into their biogenesis, roles, and release mechanisms [21,22].

Currently used techniques for the quantification of EVs include nanoparticle tracking analysis (NTA), tunable resistive pulse sensing (TRPS), flow cytometry (FCM), ZetaSizer-based Dynamic Light Scattering (DLS), surface plasmon resonance (SPR), and enzyme-linked immunosorbent assay (ELISA). NTA is a widely used method for analyzing EVs, offering a good estimate of the size distribution and concentration for EVs [23]. NTA provides real-time analysis, permitting the study of EVs in their innate state [24]; tag-free detection, decreasing possible sample preparation artifacts [25]; high sensitivity for investigating diverse vesicle populations [23]; and applicability to EVs of various sources, such as plasma and adipose tissue, upon EV isolation [23]. However, conventional NTA struggles with (a) distinguishing EVs from similarly sized lipoproteins and protein aggregates in samples [25]; (b) providing precise measurements due to the refractive index sensitivity of the particles and the nearby medium [26]; and (c) avoiding particle overlapping, often requiring high sample dilution [27].

TRPS is also a robust technique for quantifying EVs, providing a good approximation of the EV concentration with relatively low sample volumes [28,29]. By using spiked polystyrene beads during calibration, TRPS addresses some of the challenges related to measuring EVs in biological fluids, since complex biological samples can clog the pores in the TRPS system [30]. Compared to NTA, TRPS excels in detecting larger EVs (>150 nm), delivering reliable results [31]. However, like NTA, TRPS cannot discriminate EVs from lipoproteins and protein aggregates, and variations in the refractive index can impact measurement accuracy [32]. Similarly to NTA, FCM is ideal for high-throughput quantitative studies, which requires rapid data acquisition [33,34]. Different from the methods described above, FCM can specifically detect EVs and discriminate EVs from contaminants by using antibody-based fluorescence [35]. Disadvantages of conventional FCM include a low detection limit for EVs smaller than 200 nm [34], presence of artifacts due to the labeling with fluorescent antibodies or dyes [36], and mistakenly counting aggregates of dyes, antibodies, and proteins as EVs [37].

The ZetaSizer, Malvern Panalytical Ltd. (Worcestershire, UK) which uses dynamic light scattering and electrophoretic light scattering, measures the surface charge (zeta potential) of EVs and does not deliver direct quantitative data on EV concentration [38,39]. DLS measures the Brownian motion of nanoparticles in suspension and relates it to their hydrodynamic diameter based on fluctuations in scattered light intensity [40]. This method has the advantages of non-destructing EV sample—permitting for additional downstream analysis, quick analysis—facilitating the testing of various samples, and small sample volume requirement. However, like NTA and TRPS, the ZetaSizer-based DLS lacks specificity, and contaminants can bias the results as this technique presumes that all particles are sphere-shaped and alike in composition [41]. Also using optical properties, SPR is a biosensing method frequently applied to detect and quantify EVs by monitoring changes in light reflection caused by molecular interactions on a sensor surface [42]. SPR offers label-free, real-time, and highly sensitive detection of EVs (3000 exosomes/µL), enabling quantification without the need for complex sample preparation [43]. However, SPR instruments can be costly and require technical expertise, and the throughput is lower compared to some high-throughput techniques like NTA or FCM [44]. Moreover, a key disadvantage of SPR is that it requires surface functionalization with specific capture ligands, which can introduce bias toward certain EV subpopulations and limit detection to vesicles expressing the targeted surface markers [43].

Similarly, ELISA relies on antibodies that target EV surface protein markers, but EV subpopulations in a sample that have different expression levels of these markers distort the total EV count [45,46]. Therefore, this method is semi-quantitative in nature, based on protein marker levels [47]. ELISA also often requires sample purification, as contaminants in the sample can lead to false positives [48], and it is time-consuming compared to other methods as the assay requires various steps, including incubation, blocking, and washes [49]. It also highly relies on the quality of the antibodies [50]. Therefore, existing techniques for quantifying EVs have distinct drawbacks that can affect the precision, consistency, and thoroughness of their analysis. Traditional methods such as FCM, NTA, and ZetaSizer-based DLS face challenges in accurately detecting and measuring the full spectrum of EVs [51]. Advancement in EV research is further hampered by the absence of standardized protocols for quantification, isolation, and molecular analysis [11,52]. Preanalytical factors, such as sample handling, can also impact the reliability of measurements [51]. Moreover, the wide range of sizes and structural heterogeneity of EVs present challenges even for widely accepted reference techniques [53].

Due to the lack of a cost-effective and simple-to-implement method for quantifying the full spectrum of EVs with various sizes and molecular content, in this work, a paper-strip-based platform designed for efficient EV quantification using minimal sample volume and equipment was developed. This approach concentrates all EVs from a sample on the paper strip through the use of a combination of three capture antibodies (anti-CD9, CD63, and CD81), followed by membrane-stain-based fluorescence detection. ExoBrite™ dyes, which are fluorescently labeled conjugates of cholera toxin subunit B (CTB), were employed in the detection process. These dyes bind specifically to GM1 gangliosides, a lipid component commonly present on EV membranes, enabling efficient and broad-spectrum labeling of EVs [54,55]. The simplicity, scalability, and sensitivity of this method make it a promising alternative to conventional techniques. Furthermore, it holds strong potential for both basic EV research and clinical applications, addressing some of the key limitations in current EV quantification methods.

## 2. Materials and Methods

### 2.1. Materials

Whatman nitrocellulose membrane (0.45 μm pore size), TWEEN 20 viscous liquid, Amicon ultracentrifugal columns, Corning^®^ bottle top vacuum filters, and Corning^®^ low-binding tubes were purchased from Sigma Aldrich, Oakville, ON, Canada. Pierce™ Protein-free (PBS) blocking buffer (Product No. 37584), PBS 1× Ca^2+^/Mg^2+^ free, penicillin–streptomycin solution, Gibco™ human recombinant insulin, qualified fetal bovine serum, Qubit™ Protein Assay Kit, and Qubit™ Assay tubes were purchased from Thermo Fischer Scientific, Mississauga, ON, Canada. Human ovarian cancer cell line OVCAR-3, RPMI-1640 medium, normal human primary mammary epithelial (HMEC) cell line, mammary epithelial cell basal medium, mammary epithelial cell growth kit, trypsin/EDTA solution for primary cells, trypsin neutralizing solution, trypsin-EDTA 1×, purified anti-human CD9, purified anti-human CD81 (TAPA-1), and CD63 antibody (H5C6) were purchased from Cedarlane, Burlington, ON, Canada. Vent cap tissue culture flasks (non-treated, sterilized, non-pyrogenic) were purchased from VWR International, Mississauga, ON, Canada. Nitrocellulose-film slides (64 − 2.5 × 2.5 mm NC pads, 25 × 75 × 1 glass slide) were purchased from Grace Bio-Labs Inc, Bend, OR, USA. ExoBrite™ 555/575 True EV Membrane Stain (Product No. 30130) was purchased from Biotium Glowing Products for Science™, Fremont, CA, USA. The qEV size exclusion column was purchased from IZON, Medford, MA, USA.

### 2.2. Experimental Methods

#### 2.2.1. Cell Culture

OVCAR-3 human ovarian carcinoma cells were cultured in RPMI-1640 medium (ATCC formulation), enriched with 20% fetal bovine serum (*v*/*v*), 6.4 mg of human recombinant insulin, and 1% penicillin–streptomycin (*v*/*v*). Human primary mammary epithelial (HMEC) cells were grown in serum-free mammary epithelial cell basal medium (PCS-600-030) enhanced with mammary epithelial cell growth kit (PCS-600-040, Cedarlane, Burlington, ON, Canada) and 1% (*v*/*v*) penicillin–streptomycin. Complete growth cell medium for both cell lines was filtered with a 0.22 µm vacuum bottle-top filter before use. The culture flasks containing media were placed in the incubator for 30 min prior to cell seeding to allow the media to equilibrate to the incubator conditions, specifically, temperature and CO_2_ levels. This step helps ensure that the cells are introduced into an optimal and stable environment immediately upon seeding, minimizing potential stress or shock that could impact cell viability or behavior. The cells were maintained in a humidified incubator at 37 °C with 5% CO_2_, 95% air, and 90% relative humidity. Once the cell monolayer reached 80–90% confluence, they were passaged—approximately 1 × 10^6^ cells per flask.

#### 2.2.2. Size Exclusion Chromatography (SEC) EV Isolation

The supernatant from OVCAR-3 and HMEC cell cultures was concentrated 30 times using an Amicon ultrafiltration tube. A total of 15 mL of conditioned media was first centrifuged at 3000× *g* for about 15 min, then processed through an IZON qEV column to isolate EVs, following the manufacturer’s protocol. Fractions 7 through 9, which contained the highest concentration of EVs, were collected in low-protein-binding tubes and stored at −80 °C until further use. EV concentration from the SEC-purified sample was estimated to be approximately 1.1 × 10^9^ particles/mL using ZetaSizer-based DLS analysis (Malvern Panalytical Ltd., Worcestershire, UK)—measurement based on dynamic light scattering (DLS).

#### 2.2.3. EV Total Protein Concentration Measurement

QuBit Fluorometer was used to measure the total protein concentration of the SEC-purified EV samples. To prepare the Qubit working solution, the Qubit Protein Reagent was diluted 1:200 in Qubit Protein Buffer according to the manufacturer’s instructions. The solution was gently mixed by pipetting up and down to avoid bubble formation. For measurement, 180 µL of the working solution was dispensed into Qubit assay tubes, followed by the addition of 20 µL of EV samples. Each tube was briefly mixed by pipetting and incubated at room temperature for 15 min, shielded from light. Following incubation and after calibration with the three QuBit protein standards, sample tubes were inserted one at a time, and the total protein concentration (µg/mL) was recorded.

#### 2.2.4. Immunoassay Procedure for the Estimation of EV Concentration

**Figure 1:**
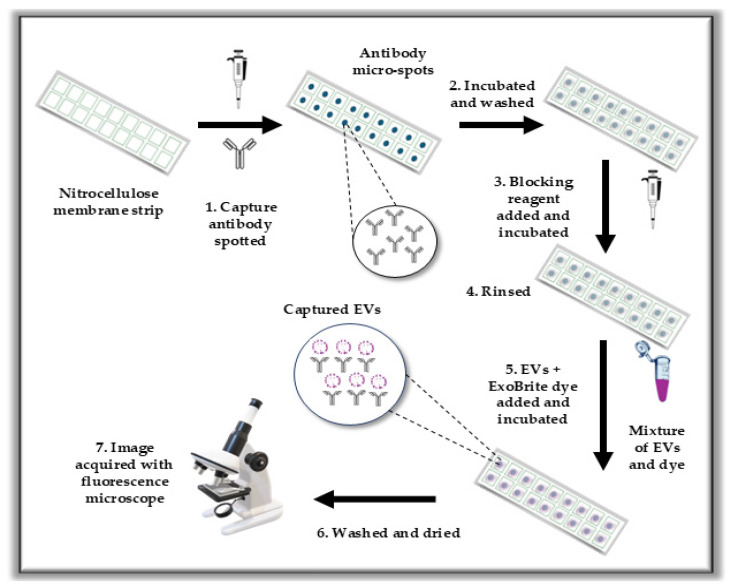
Schematic of the immunoassay procedure for the capture and estimation of extracellular vesicles on nitrocellulose.

The immunoassay used to estimate the EV counts from the cell line’s media is depicted in Figure 1. All experiments were performed with eukaryotic EVs derived from human cell lines. To perform the immunoassay, anti-CD63 (17 μg/mL), anti-CD9 (17 μg/mL), and anti-CD81 (17 μg/mL) antibodies prepared in filtered 1× DPBS with 10% glycerol were applied to the nitrocellulose (NC)-film slides or NC strips. Glycerol was added to DPBS to prevent the capture antibody from drying on the NC membrane during spotting. Drying can affect the structural integrity and functional orientation of the antibody, potentially compromising its ability to capture the target antigen efficiently. The spotting volume was 0.4 µL per spot for the NC-film slides and 0.5 µL per spot for the membrane. NC membranes were trimmed into ~5 × 2 cm rectangles and adhered to plain glass slides using tape for imaging (last step). NC-film slides were sourced commercially.

Following spotting, the substrates were placed in a humidified chamber and incubated for 1 h at room temperature in the dark. They were then washed three times with DPBS. The NC surface was then treated with a ready-to-use blocking formulation (Pierce™ Protein-free blocking buffer) and incubated for 1 h at room temperature, followed by a brief DPBS rinse. Only 1 µL of the ready-to-use blocking formulation was applied per square to minimize the risk of cross-contamination or leakage into neighboring sample regions on the membrane. This step was essential to minimize nonspecific binding and improve the assay’s signal-to-noise ratio.

After optimization, ExoBrite™ True EV Membrane staining solution was prepared by diluting the dye stock 1:200 in filtered DPBS. Then 450 µL of the ExoBrite™ staining solution was added to the tube containing 50 µL of EVs or control (DPBS). Immediately after blocking, EVs labeled with the ExoBrite™ staining solution were added to the substrate and incubated for 20 min in the dark at room temperature. After incubation, the substrate was washed three times with PBST 0.05%. The NC membrane was then dried using a gentle stream of air applied with an air-blower. This step was performed after the final wash and just before imaging, to remove any residual liquid on the membrane surface and ensure a uniform, dry background for accurate fluorescence signal detection. Finally, the NC membrane was imaged using a fluorescence microscope.

#### 2.2.5. Signal Acquisition and Data Analysis

The samples were imaged with the Nikon Eclipse Ti fluorescence microscope, Nikon Corporation, Melville, NY, USA (1280 × 1024 no binning, 1.0× analog gain, exposure time: 900 msec for the NC-film slides, and 300 msec for the membrane) using a red excitation filter (wavelength of 556 to 576 nm). ImageJ1.54g software was used to obtain fluorescent intensities of the spots. For all images, mean gray values were used for the signal quantification. MATLAB R2022b software was used for statistical analysis and for graphing the results. ANOVA and Student *t*-test for independent samples were used to detect significant differences in the fluorescent intensities between conditions.

## 3. Results

### 3.1. Optimizing Dye Application Sequence to Enhance EV Detection Sensitivity

**Figure 2:**
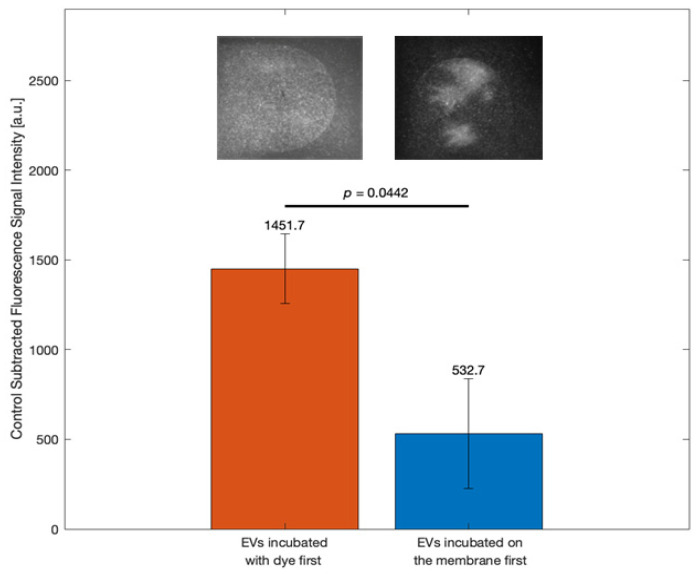
Comparison of signals for different EVs/CTL incubation procedure with the dye and the nitrocellulose membrane. The mean of the control was subtracted. Data represent mean ± standard error (*n* = 3).

Figure 2 presents a bar graph comparing fluorescence signal intensities for two experimental procedures: (1) EVs were mixed with the ExoBrite™ staining solution first, and then the EV/dye mixture was incubated on the membrane (red bar), and (2) EVs were first incubated on the membrane before the addition of the ExoBrite™ staining solution (blue bar). The Y-axis represents the fluorescence signal intensity from the EV samples, deducted from the control signal intensity (filtered 1× DPBS), in arbitrary units (a.u.), while the X-axis shows the two conditions. The red bar (EV sample mixed with ExoBrite™ stanning solution and then incubated on the membrane) showed a significantly higher fluorescence signal (1451.7 a.u.) compared to the blue bar (532.7 a.u.). The error bars reflected variability, but with no overlap, indicating a clear separation between the mean fluorescence values. The *p*-value (*p* = 0.0442) confirmed a statistically significant difference between the two procedures. Fluorescence images above the bars visually supported the signal difference, showing a stronger and more homogenous signal for the first procedure. This optimization step confirmed that pre-mixing EVs with the staining solution prior to membrane incubation enhances assay sensitivity by improving labeling efficiency and signal consistency.

### 3.2. Determining Optimal Incubation Duration for Enhanced EV Fluorescence Signal

As outlined by the supplier [56], the ExoBrite™ True EV Membrane staining solution was prepared by diluting the 500X stock solution using filtered PBS. To stain samples, 450 µL of the prepared 1X staining solution was added to each tube containing 50 µL of EV sample—1.1 × 10^9^ EV particles/mL. The same staining solution was also added to the control tube containing only filtered PBS. Although the supplier’s protocol recommends a 30 min incubation time, it was necessary to optimize this parameter for our specific experimental setup. In contrast to the manufacturer’s suggested procedure, where the dye is incubated directly with EVs in solution (i.e., in a tube), our approach involved applying the dye–EV mixture directly onto an NC membrane. This change in context required us to evaluate the optimal incubation time on the membrane surface, as the dynamics of dye–EV interaction and fluorescence signal development could differ significantly from those in solution. Therefore, a time-course experiment was conducted to determine the incubation duration that yields the strongest and most reliable fluorescence signal under our membrane-based assay conditions.

**Figure 3:**
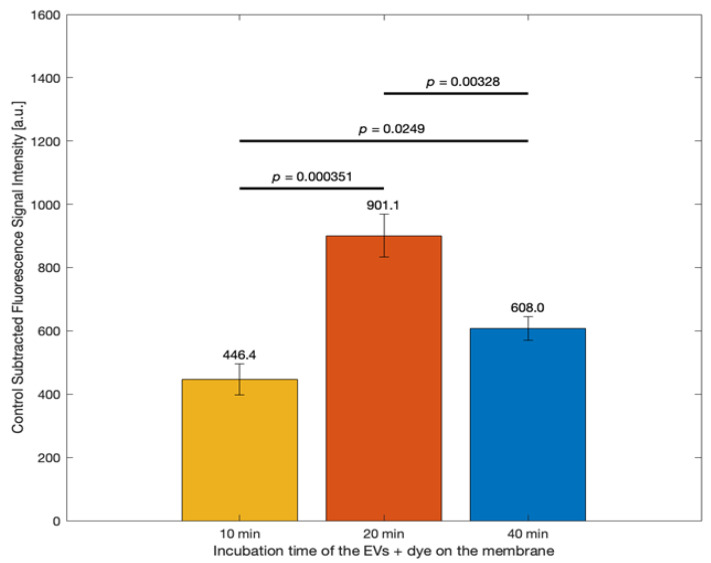
Comparing signals from the EVs/dye mixture incubated on the membrane for distinct duration periods (10, 20, and 40 min)—mean control subtracted. Data represent mean ± standard error (*n* = 3).

Figure 3 displays the graph comparing the fluorescence signal intensities from EVs in the ExoBrite™ staining solution incubated on the membrane for different durations: 10, 20, and 40 min. The Y-axis shows control-subtracted fluorescence intensity (a.u.), and the X-axis categorizes the incubation times. The highest signal was observed with a 20 min duration (901.1 a.u.), followed by 40 min (608.0 a.u.) and 10 min (446.4 a.u.). Statistical significance was indicated between the groups, with *p*-values provided for each pairwise comparison. The data revealed that a 20 min incubation yielded the strongest fluorescence signal, nearly doubling that of the 10 min condition and significantly surpassing the 40 min group. Statistical tests (*p* < 0.05 for all comparisons) confirmed that these differences were significant. A 20 min incubation was identified as the optimal duration for EV/dye interaction on the membrane, yielding the highest fluorescence signal, while longer incubation (40 min) led to reduced signal, likely due to over-incubation effects such as dye degradation or EV detachment.

### 3.3. Evaluation of Single vs. Multi-Antibody Capture Strategies for EV Detection

**Figure 4:**
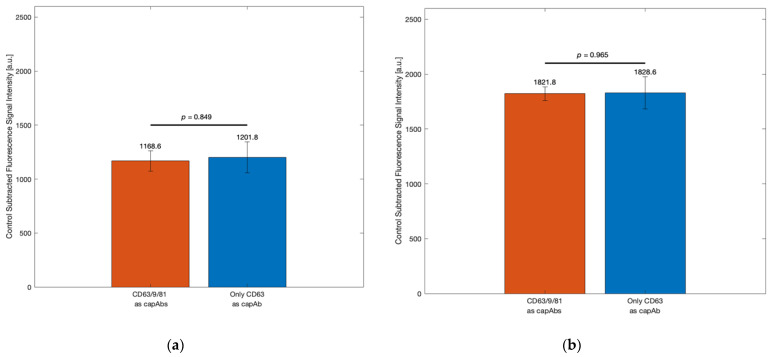
Optimization of the capture antibody—one or three antibodies: (**a**) OVCAR cell line capture antibody optimization and (**b**) HMEC cell line capture antibody optimization. Data represent mean ± standard error (*n* = 3).

Figure 4 shows two bar graphs (a and b) comparing fluorescence signal intensities for two antibody capture strategies: using anti-CD63/9/81 (three antibodies) vs. anti-CD63 alone (one antibody). CD9, CD63, and CD81 are widely used as markers for EVs due to their high abundance on the EV surface membrane. These tetraspanin proteins are encoded by genes located on human chromosomes 11 and 12—specifically, CD9 at 12p13.31, CD63 at 12q13.2, and CD81 at 11p15.5 [57]. A combination of anti-CD63, anti-CD9, and anti-CD81 was chosen to bind the CD63, CD9, and CD81 proteins on the EV surface membrane because these proteins are known to be commonly expressed at different concentrations across other cell types or EV subpopulations [58,59]. Figure 4a represents results from the SEC-isolated EVs from OVCAR3 cancer line medium, and Figure 4b from the SEC-isolated EVs from HMEC epithelial cell line medium. The differences were not statistically significant between the two approaches, as indicated by the *p*-values (*p* = 0.849 in a, *p* = 0.965 in b). Error bars overlapped in both comparisons, indicating similar capture efficiency. The results indicated that while anti-CD63 alone was sufficient for capturing EVs from OVCAR3 and HMEC cell lines, using a combination of anti-CD63, CD9, and CD81 antibodies may enhance capture efficiency and EV population representativeness in more heterogeneous or CD63-deficient samples.

### 3.4. Optimization of ExoBrite™ Dye Concentration to Enhance EV Detection Sensitivity

**Figure 5:**
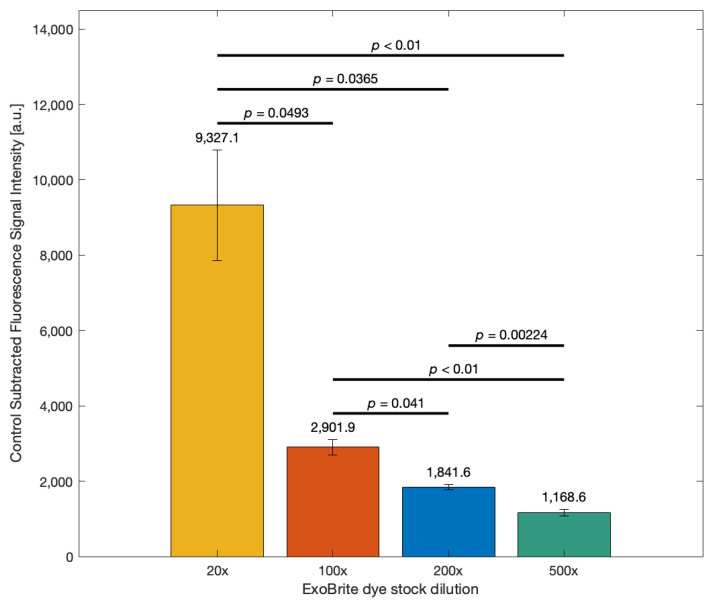
Effect of the ExoBrite™ dye concentration on fluorescence signal intensity. EV samples were labeled with the staining solution prepared at different dilutions (20×, 100×, 200×, and 500×), and the fluorescence signal from the control (DPBS) was subtracted from the one measured for the sample. Data represent mean ± standard error (*n* = 3).

Figure 5 illustrates the effect of different dye concentrations (dye stock solution was diluted 20×, 100×, 200×, and 500×) on the control-subtracted fluorescence signal intensity (a.u.) from SEC-isolated EVs from OVCAR3 cancer line medium. Here, the x-axis lists the dilution factors. The highest signal was observed at 20× dilution (9327.1 a.u.), followed by a significant drop at 100× (2901.9 a.u.), a further decline at 200× (1841.6 a.u.), and the lowest signal at 500× dilution (1168.6 a.u.). Statistically significant differences between all groups were indicated by the respective *p*-values (*p* < 0.05). The fluorescence intensity increased sharply with decreasing dilution of the ExoBrite™ dye. The 20× dilution condition resulted in over threefold higher signal compared to 100× dilution, nearly fivefold higher than 200× dilution, and almost tenfold higher than 500× dilution. The 200× dilution condition offered a more stable and reproducible signal with minimal background interference, making it the optimal choice for this assay.

These findings are consistent with recent studies that have explored ExoBrite™ dye performance across varying concentrations. Brealey et al. tested 50×, 25×, 10×, 5×, 2.5×, 1×, 0.5×, 0.25×, and 0.1× dilutions of ExoBrite™ 640/660 for nano-flow cytometry analysis of SW480-derived EVs [55]. They evaluated ExoBrite™ alongside other EV labeling dyes and found that optimized low dilutions of ExoBrite™ provided high fluorescence positivity with minimal background signal in nano-flow cytometry applications, reinforcing the importance of dilution optimization for maximizing sensitivity while avoiding dye aggregation [55]. Similarly, Kozela et al. applied ExoBrite™ 560 diluted 100× in spectral flow cytometry to detect DNA cargo within malaria parasite-derived EVs and emphasized the importance of dye titration to distinguish subpopulations of labeled vesicles accurately [60]. These findings highlight the critical role of dye concentration optimization for effective EV detection in fluorescence-based assays. However, the optimal dye concentration for membrane-based assays differs from flow cytometry-based applications. Unlike flow cytometry systems, NC membranes exhibit inherent autofluorescence, which influences the background signal and necessitates tailored optimization.

### 3.5. Estimating Different EV Concentrations with the EV Paper Strip

**Figure 6:**
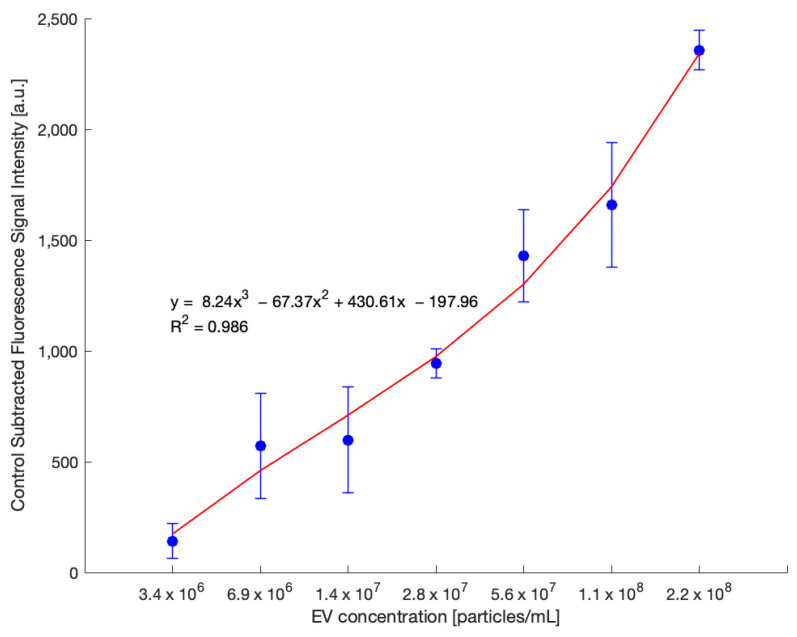
Standard curve generated using a twofold serial dilution of SEC-isolated EVs from OVCAR3 cell culture medium. EV concentrations ranged from 2.2 × 10^8^ down to 3.4 × 10^6^ particles/mL, and control-subtracted fluorescence signal intensities were measured. A concentration-dependent increase in fluorescence was observed, demonstrating the assay’s capability to quantify EVs across a defined dynamic range. Data are shown as mean ± standard error (*n* = 3).

Next, the quantitative range and detection sensitivity of the EV fluorescence assay were evaluated. Figure 6 presents a standard curve generated using a twofold serial dilution of SEC-isolated EVs from OVCAR3 cell culture medium. As presented in Figure 6, the x-axis represents EV concentrations ranging from 3.4 × 10^6^ to 2.2 × 10^8^ particles/mL, while the y-axis displays the corresponding fluorescence signal intensity (in arbitrary units), subtracted from the background signal. Each point represents the average of replicate measurements, and error bars denote the standard error.

A clear positive correlation between EV concentration and fluorescence signal was observed. The data followed a nonlinear trend, best described by a third-degree polynomial regression model: y = 8.24x^3^ − 67.37x^2^ + 430.61x − 197.96, with an excellent coefficient of determination (R^2^ = 0.986). This strong correlation validates the assay’s ability to detect incremental increases in EV concentration. The signal increased consistently across the full concentration range, particularly at mid to high EV levels, indicating the assay’s suitability for semi-quantitative analysis.

### 3.6. Validation Using Total Protein Quantification

**Figure 7:**
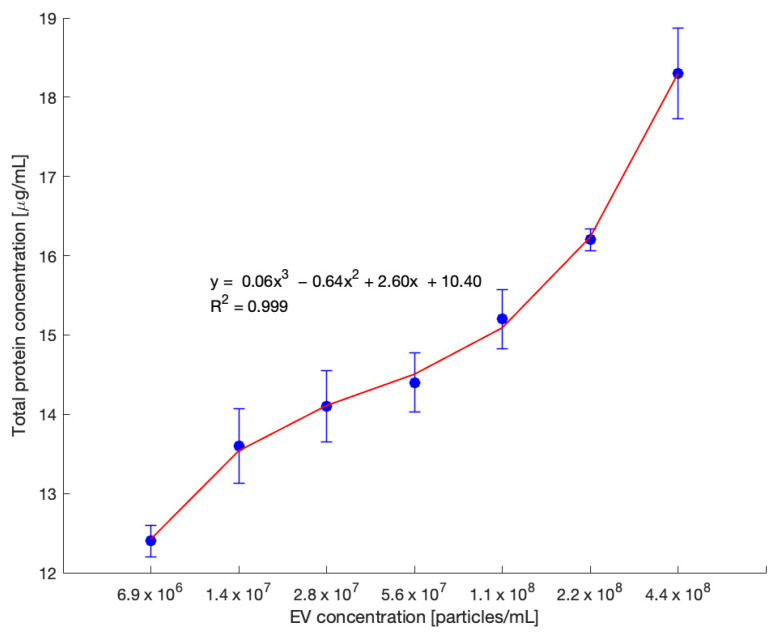
Standard curve for the total protein concentration generated using the same twofold serial dilution of SEC-isolated EVs from OVCAR3 cell culture medium as in Figure 6. EV concentrations ranged from 4.4 × 10^8^ down to 6.9 × 10^6^ particles/mL, and total protein concentrations in µg/mL were measured. A concentration-dependent increase in total protein concentration was observed, demonstrating a consistent trend with the developed quantification technique, supporting the reliability of our measurements (*n* = 3).

To further validate the performance of the developed fluorescence-based EV quantification assay, total protein content was measured using the Qubit fluorometric assay on the same set of EV samples. These samples were subjected to a twofold serial dilution, consistent with the conditions used in the fluorescence assay (Figure 6), to enable direct comparison. As shown in Figure 7, the x-axis represents EV concentration in particles/mL, and the y-axis displays the corresponding total protein concentration (µg/mL) for each dilution.

The resulting data exhibited a strong, nonlinear correlation between EV concentration and total protein content. A third-degree polynomial regression was applied to fit the data, yielding the equation y = 0.06x^3^ − 0.64x^2^ + 2.60x + 10.40, with a high R^2^ value of 0.999, indicating an excellent fit. Each point in the graph represents the mean of replicate protein measurements, and error bars reflect the standard error. The clear trend observed across the dilution series reinforces the consistency of the protein yield relative to EV concentration.

## 4. Discussion

The optimization study in Section 3.1 demonstrated that the sequence of dye application significantly impacts assay sensitivity. As shown in Figure 2, pre-mixing EVs with the ExoBrite™ staining solution before incubation on the membrane generated a substantially stronger fluorescence signal compared to sequential incubation. The absence of overlapping error bars and a statistically significant *p*-value (*p* = 0.0442) indicate that this difference is unlikely due to random variation. These findings suggest that thorough mixing of EVs with dye molecules by pipetting up and down improves labeling efficiency and contributes to more robust and consistent detection.

In Section 3.2, we further optimized assay conditions by evaluating the incubation time of the EV/dye mixture on the membrane. Although the manufacturer’s protocol recommends a 30 min incubation in solution, our membrane-based format required re-evaluation. Figure 3 shows that a 20 min incubation produced the highest fluorescence signal, while longer incubation (40 min) resulted in signal decline, likely due to dye degradation or EV detachment. These results suggest that a 20 min incubation provides an optimal balance between signal strength and stability for membrane-based detection, though further investigation may be warranted to understand the causes of over-incubation effects.

Section 3.3 examined the impact of antibody configuration on EV capture. The results from Figure 4, based on EVs from OVCAR3 and HMEC cell lines, showed no significant difference between capture using anti-CD63 alone and a combination of anti-CD63, CD9, and CD81 antibodies. This is likely due to the relatively high expression of CD63 in EVs from these specific cell types. Supporting this observation, Momenbeitollahi et al. [61] successfully employed CD63 as the sole capture antibody in a dot blot assay and demonstrated a robust ability to capture EVs derived from the OVCAR-3 cell line. The assay detected multiple downstream surface markers (CD9, CD24, EpCAM) with high sensitivity—down to 4.7 × 10^4^ EVs/mL—confirming that CD63 effectively concentrates the majority of EV subtypes from OVCAR-3 cells. Similarly, Goodrum and Li [62] conducted multiplexed reverse-phase immunoassays targeting EV surface and intravesicular markers (CD9, EGFR, HSP70) from OVCAR-3-derived EVs and reported strong fluorescence signals and low detection limits across all markers after lysis. The consistent detectability of multiple proteins, both on the EV surface and within, strongly suggests that the OVCAR-3 EV population broadly expresses these recognized tetraspanins, and by extension, that CD63 can serve as a sufficient capture marker in some contexts.

However, in other biological contexts, tetraspanin expression can vary significantly across different EV subtypes and cellular sources. For example, studies have shown that CD63 is not uniformly expressed in all EV populations, with some EVs—depending on the cell type and physiological context—exhibiting low or undetectable levels of CD63 [63,64]. Consequently, relying solely on CD63 may lead to incomplete EV recovery by excluding subpopulations that lack this marker. In contrast, using a combination of anti-CD63, CD9, and CD81 antibodies can improve the overall enrichment and representativeness of EVs captured on the membrane by targeting a broader range of surface markers across heterogeneous EV populations.

Section 3.4 identified dye concentration as a key parameter influencing detection sensitivity. As shown in Figure 5, the 500× dilution showed the lowest fluorescence signal intensity among all tested dye concentrations. This reduction is likely due to insufficient dye availability to label the EVs effectively, leading to a weak signal. While this condition exhibited low variability across replicates, the signal was too low to be considered optimal for EV detection and quantification. At this high dilution, the assay risks underestimating EV presence due to limited dye incorporation, thereby compromising sensitivity.

Although the 20× dilution produced the highest fluorescence signal intensity, it was not selected as the optimal condition due to two key concerns. First, the high fluorescence intensity approached the upper limit of the detector’s dynamic range, introducing the risk of signal saturation. Signal saturation can impair the assay’s ability to detect subtle differences between samples, as the fluorescence detector may no longer respond linearly to increases in signal, leading to quantitative inaccuracies and compromised reproducibility [65]. Second, the large error bar observed at 20× dilution indicated substantial variability between replicates, suggesting potential inconsistencies in signal generation at this dye concentration. High dye concentrations can increase non-specific binding, reducing signal-to-noise ratio and potentially producing false positives. This means that small differences in EV concentrations between the samples can no longer be detected.

The 100× dilution was also excluded due to its less distinct separation from the control signal. Ensuring a clear separation between sample and control signals is critical for reproducibility, especially when scaling the assay for comparative or clinical studies. In contrast, the 200× dilution condition demonstrated a favorable balance between signal intensity, reproducibility, and low background. Therefore, 200× dilution was selected as the optimal working concentration for ExoBrite™ in this assay.

The standard curve in Figure 6 confirmed the assay’s ability to detect incremental changes in EV concentration across a defined range. The twofold serial dilution approach successfully produced a standard curve suitable for semi-quantitative analysis of EVs. The clear concentration-dependent increase at mid to high EV concentration highlighted the assay’s detection range. The large error bars observed likely stem from cumulative variability introduced by manual pipetting steps throughout the experimental protocol. Each handling step—such as dilution, incubation, and washing—can contribute to variability in fluorescence measurements. Additionally, sample stability may have influenced signal consistency. Implementing automated liquid handling systems in future work could improve assay precision and reproducibility.

Lastly, the protein-based standard curve serves as an independent confirmation of the fluorescence-based quantification method. The strong correlation across both assays supports the validity of using total protein concentration as a proxy for EV abundance and highlights the reproducibility of the developed platform. Together, these results demonstrate that the fluorescence signal trends align closely with total protein measurements, providing robust evidence for the assay’s reliability in estimating EV levels within a defined quantitative range.

## 5. Conclusions

The development of an NC-based strip for estimating EV counts offers a streamlined, accessible alternative to conventional EV quantification methods. Compared to current standard techniques such as NTA, TRPS, SPR, and ELISA, which each have notable limitations—ranging from low throughput and sample preparation artifacts to low sensitivity and specificity—the proposed assay simplifies workflow while maintaining quantification capacity and adaptability to various sample types.

Optimization experiments were essential in maximizing the assay’s performance. First, the sequence of dye and EV incubation time was found to significantly influence fluorescence signal intensity. Pre-labeling EVs in the ExoBrite™ solution before membrane application resulted in a stronger and more consistent signal compared to sequential incubation, likely due to more efficient dye binding or membrane interaction. Additionally, a 20 min incubation period for the EV–dye mixture on the membrane was identified as optimal, balancing signal strength and reproducibility while avoiding potential dye degradation or vesicle detachment observed at longer durations.

Interestingly, no significant improvement in signal was observed when using a combination of three capture antibodies (CD63, CD9, and CD81) compared to CD63 alone, with the two cell lines that were tested. This finding is important for assay simplification and cost reduction, as it suggests that CD63 alone may be sufficient for EV capture in certain contexts. Nonetheless, it is important to acknowledge that tetraspanin expression can vary across cell types and EV subpopulations. Thus, marker selection should be carefully tailored to the specific biological source or diagnostic application, and reliance on a single tetraspanin marker like CD63 may not be universally sufficient for all cell lines or sample types.

A critical parameter affecting sensitivity was the concentration of ExoBrite™ dye. Although lower dilutions (e.g., 20×) resulted in markedly higher signals, they also introduced the risk of signal saturation [65], background noise, and non-linearity in quantification. A 200× dilution provided the best compromise between sensitivity and specificity, avoiding detector saturation and maintaining a linear dynamic range suitable for semi-quantitative analysis.

The standard curve generated through twofold serial dilution of SEC-isolated EVs confirmed the assay’s quantification capability. A strong positive correlation between EV concentration and fluorescence intensity was observed within the mid to high concentration range, establishing a usable dynamic range, which aligns with most biological samples containing EVs. However, high variability in signal at lower concentrations suggests limitations in sensitivity for detecting low-abundance EV populations. Further refinements—such as improved imaging parameters or membrane formulations—may help reduce noise and increase detection fidelity in this range.

The developed paper-strip platform offers a simplified, accessible, and cost-effective approach for EV quantification, contrasting with conventional techniques such as NTA, TRPS, FCM, ZetaSizer-based DLS, SPR, and ELISA. Unlike these instrument-heavy methods, the paper-strip assay operates with minimal sample volumes, requires no complex instrumentation, and utilizes a combination of anti-CD9, CD63, and CD81 capture antibodies along with membrane-stain-based fluorescence detection. This setup enables semi-quantitative EV analysis in a format that is highly suitable for low-resource settings and point-of-care applications.

While NTA and TRPS provide valuable data on particle size distribution and concentration, they require technical expertise, calibration, and relatively large sample volumes (300–1000 µL for NTA and 40–100 µL for TRPS), and they lack molecular specificity—struggling to distinguish EVs from similarly sized contaminants [30,32,66,67]. FCM and ELISA offer high sensitivity and marker-specific detection but are time-consuming, dependent on expensive reagents and instruments, and require skilled operators [68,69,70,71]. The ZetaSizer-based DLS, although effective for measuring EV size and zeta potential, lacks the ability to differentiate EVs from protein aggregates and other nanoparticles in biological samples [41,72]. Similarly, SPR offers label-free, real-time kinetic analysis but is limited by high operational costs and the need for technical expertise [73,74]. In contrast, the paper-strip platform balances sensitivity, simplicity, and specificity, making it a highly practical tool for rapid EV detection where comprehensive profiling is not essential.

Overall, the paper-strip-based platform developed in this study demonstrates significant potential for rapid, low-cost, and scalable EV quantification. Its compatibility with minimal equipment and small sample volumes makes it especially promising for applications in clinical diagnostics and point-of-care testing. Future work may focus on expanding the assay’s multiplexing capabilities, enhancing detection sensitivity at the lower range, and validating performance across diverse biological fluids and disease models [75,76].

## Data Availability

Data are available upon request.

## Abbreviations

The following abbreviations are used in this manuscript:

EVs	Extracellular vesicles
DLS	Dynamic Light Scattering
SEC	Size exclusion chromatography
SPR	Surface plasmon resonance
NTA	Nanoparticle tracking analysis
TRPS	Tunable resistive pulse sensing
FCM	Flow cytometry
ELISA	Enzyme-linked immunosorbent assay
NC	Nitrocellulose
